# Chloroquine neither eliminates liver stage parasites nor delays their development in a murine Chemoprophylaxis Vaccination model

**DOI:** 10.3389/fmicb.2015.00283

**Published:** 2015-04-09

**Authors:** Tejram Sahu, Lynn Lambert, Jessica Herrod, Solomon Conteh, Sachy Orr-Gonzalez, Dariyen Carter, Patrick E. Duffy

**Affiliations:** Laboratory of Malaria Immunology and Vaccinology, National Institute of Allergy and Infectious Diseases, National Institutes of Health, RockvilleMD, USA

**Keywords:** *Plasmodium*, chloroquine, liver-stage, CVac, prepatent

## Abstract

Chemoprophylaxis Vaccination (CVac) confers long lasting sterile protection against homologous parasite strains in humans, and involves inoculation of infectious sporozoites (SPZ) under drug cover. CVac using the drug chloroquine (CQ) induces pre-erythrocytic immunity in humans that includes antibody to SPZ and T-cell responses to liver stage (LS) parasites. The mechanism by which CVac with CQ induces strong protective immunity is not understood as untreated infections do not confer protection. CQ kills blood stage parasites, but its effect on LS parasites is poorly studied. Here we hypothesized that CQ may prolong or perturb LS development of *Plasmodium*, as a potential explanation for enhanced pre-erythrocytic immune responses. Balb/c mice with or without CQ prophylaxis were infected with sporozoite forms of a luciferase-expressing rodent parasite, *Plasmodium yoelii*-Luc (*Py*-Luc). Mice that received primaquine, a drug that kills LS parasites, served as a positive control of drug effect. Parasite burden in liver was measured both by bioluminescence and by qRT-PCR quantification of parasite transcript. Time to appearance of parasites in the blood was monitored by microscopic analysis of Giemsa-stained thick and thin blood smears. The parasite load in livers of CQ-treated and untreated mice did not significantly differ at any of the time points studied. Parasites appeared in the blood smears of both CQ-treated and untreated mice 3 days after infection. Taken together, our findings confirm that CQ neither eliminates LS parasites nor delays their development. Further investigations into the mechanism of CQ-induced protection after CVac are required, and may give insights relevant to drug and vaccine development.

## Introduction

The most successful vaccination approach against malaria involves immunization with whole organism, generally by the liver-infective form called sporozoites (SPZ). SPZ attenuated by radiation (RAS; Nussenzweig et al., 1967; Clyde et al., 1973) or by genetic alterations (GAP; Mikolajczak et al., 2010; Vaughan et al., 2010) infect hepatocytes but arrest during liver stage (LS) development and do not emerge in the blood stream. More recently, experimental infection and drug treatment has achieved impressive protection: in humans, inoculation of SPZ under drug cover, referred to here as Chemoprophylaxis Vaccination (CVac), required much lower SPZ doses than RAS to induce sterile immunity that persisted for >2 years in four of six individuals (Belnoue et al., 2004; Roestenberg et al., 2011). Chloroquine (CQ) prophylaxis has been studied most extensively as drug cover during CVac, and this regimen generates preerythrocytic immunity that includes anti-sporozoite antibody (Behet et al., 2014) and LS-specific T-cells in animals and humans (Belnoue et al., 2004; Renia, 2008; Bijker et al., 2013).

Chloroquine kills blood stage parasites as they reach the trophozoite stage of development (Yayon et al., 1983), but the effect of CQ on LS parasites has received less attention. It is widely believed that CQ does not affect LS development, allowing the immune system to encounter the full repertoire of LS antigens. However, the more durable protection achieved with CVac after ∼16-fold fewer mosquito bites than required for RAS suggests that drug treatment effects might contribute independently to protection.

The effect of CQ on *Plasmodium* LS development is not completely settled. Based on an *in vivo* infection model, Belnoue et al. (2004) reported that CQ does not kill *Plasmodium* LS at 42 h post infection with SPZ. However, the effect of CQ on later time points including blood stage emergence has not been studied. Using an *in vitro* infection model, Fisk et al. (1989) reported a partial schizonticidal effect of CQ on late LS, but the CQ dose was relatively high, and had cytotoxic effects on hepatocytes as well. Here we report a systematic evaluation of the effects of therapeutic concentrations of CQ on late LS development, using a luciferase expressing *P. yoelii* parasite in Balb/c mice.

## Materials and Methods

### Mice

Female Balb/c mice, 4–6 week old were purchased from Taconic. Mice were housed in the NIH animal facility under pathogen free conditions and fed with autoclaved food *ad libitum*. All experiments were performed once mice reached 16 to 18 weeks of age. All experiments were approved and performed as per the guidelines by the Animal Care and Use Committee (ACUC) of NIAID/NIH.

### Parasite and Mosquitoes

*Plasmodium yoelii* parasites (strain 17XNL), including the parental line and a transgenic line expressing firefly-luciferase and GFP (*Plasmodium yoelii*-Luc, *Py*-Luc), were maintained by cycling between Balb/c mice and *Anopheles stephensi* mosquitoes. Salivary gland SPZs were harvested on days 14–18 as described earlier (Ozaki et al., 1984; Guebre-Xabier et al., 1999).

### Infection and Evaluation of LS Development by Bioluminescence Imaging

Female Balb/c mice (16–18 weeks old) were infected with 15,000 freshly dissected SPZ of *Py*-Luc in 100 μl of 1X PBS+2% normal mouse serum by IV injection. Development of LS was monitored by bioluminescence imaging (BLI) at different time points post-infection as described earlier (Mwakingwe et al., 2009; Miller et al., 2013). Briefly, mice were injected intradermally with 150 μl of Rediject D-Luciferin (Caliper Life Sciences, USA), then anesthetized with isoflurane-anesthesia system 8–10 min later to allow measurement of bioluminescence and image acquisition using IVIS-100 animal imager (Caliper Life Sciences, USA). Bioluminescence images were acquired at the following settings: 15 cm Field of View (FOV), medium binning, and exposure time of 30–60 s. Quantification of luminescence was performed using the ROI settings of the Living Image 4.4 software (Perkin Elmer, USA). ROI were drawn around the abdominal area locating the liver. ROI measurements were expressed as total flux photons per second (p/s).

### Drug Treatment and LS Burden Estimation by Intravital Imaging

Infected mice comprised three groups that received different treatments in 100 μl of PBS by intraperitoneal (IP) route: one group received 0.8 mg/mouse of CQ diphosphate (SPZ+CQ; Sigma; Belnoue et al., 2004): one group received 1.5 mg/mouse of PQ bisphosphate (SPZ+PQ; Sigma; Putrianti et al., 2009); one group received PBS without drug (SPZ). Two drug control groups were also included, whereby mice received CQ or PQ but no SPZ infection. An additional control group received no drug and no SPZ. Drug was delivered at the time of infection (0 hours post infection, hpi) and at 24 hpi. Bioluminescence images were acquired *in vivo* with IVIS-100 (Perkin Elmer) at different time points after drug injection indicated in the figure legends. BLI was performed on whole livers that had been perfused (10 ml of RNAse free 1x PBS) and isolated at sequential time points from 40 hpi onward. Liver samples were also snap-frozen in liquid nitrogen for qRT-PCR.

### Determination of LS Burden by qRT-PCR

Total RNA was extracted from the whole liver as described earlier (Schussek et al., 2013) using RNeasy mini kit (Qiagen Inc). cDNA was synthesized using High-Capacity cDNA Reverse Transcription Kit (Applied Biosystem, Foster City, CA, USA). Gene expression was measured with 1:40 dilutions of cDNA. Standard curve quantitative RT-PCR was performed (Bruna-Romero et al., 2001) in a 20 μl volume, which includes 1X ABI Power SYBR master mix (Applied Biosystems) and 0.25 μM of either *P. yoelii* 18S rRNA primer (forward- GGGGATTGGTTTTGACGTTTT, reverse- AAGCATTAAATAAAGCGAATA) or mouse β-actin primers (Forward- GGCTGTATTCCCCTCCAT; reverse-CCAGTTGGTAACAATGCAAT). PCR reactions were run on ABI 7500 machine (Applied Biosystems), using the following conditions: 50°C for 2 min; 95°C for 10 min; 40 cycles of 95°C for 15 s alternating with 60°C for 1 min.

cDNA standards for both 18S rRNA and β-actin were prepared as 10-fold dilutions (10^7^–10^3^ copies) from purified PCR product. Liver of naïve mouse was used as negative control. Parasite load was normalized to host β-actin as a ratio (absolute copy of *Py* 18S/ absolute copy of mouse β-actin).

### Determination of Prepatent Period

Thick and thin blood smears were collected from the infected mice at different time points starting 42 hpi. Blood smears were Giemsa-stained and examined with a bright field microscope with 100× oil-immersion objective and by expert slide readers blinded to the study groups. Blood smears were considered positive if at least two infected RBCs were found in 100 adjacent fields.

### Statistical Analysis

Mann–Whitney test was used to compare groups for LS burden measured by either BLI or qPCR. *P* ≤ 0.05 was considered statistically significant. GraphPad Prism software (version 6) was used for statistical analysis.

## Results

### LS Parasites Persist 54 H After SPZ Inoculation into Untreated Mice

**FIGURE 1:**
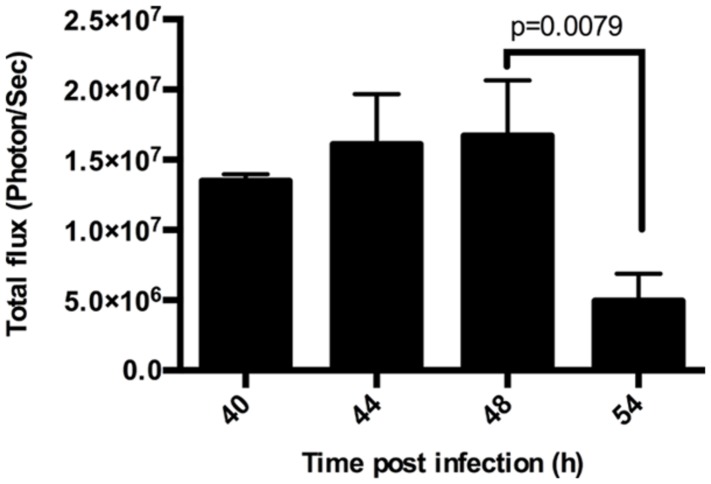
**Duration of *Plasmodium yoelii*-Luc (*Py*-Luc) liver stage (LS) growth.** Graph representing quantification of total flux from infected and untreated mice imaged at 40, 44, 48, and 54 h after sporozoites (SPZ) injection. *n* = 3 for 40 h, 4 for 44 h, and 5 each for 48, and 54 h. Graph represents Mean ± SD. Mann–Whitney test was performed and *p* < 0.05 considered as significant.

**FIGURE 2:**
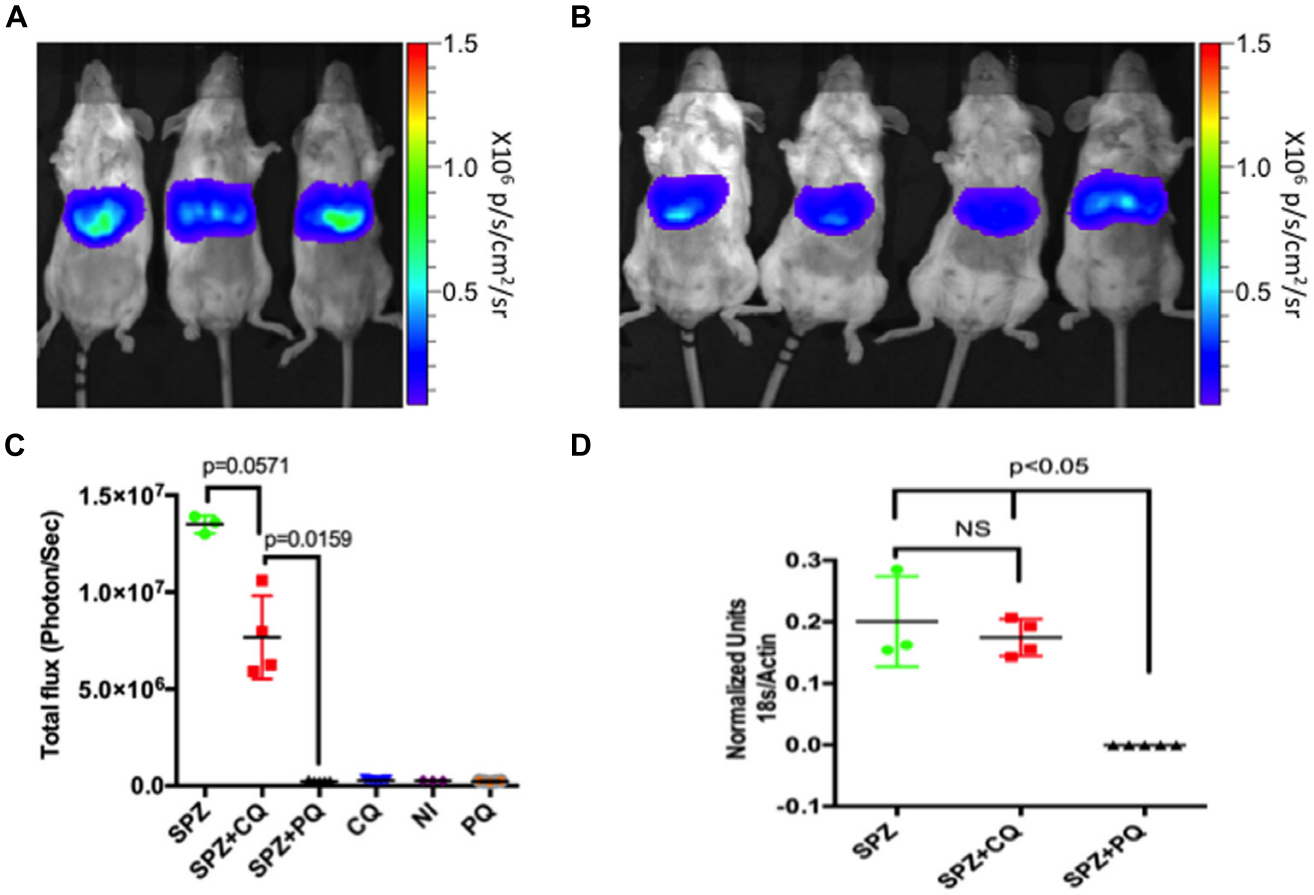
**Effect of CQ on LS parasite at 40 hours post infection (hpi).** Rainbow images of mice infected and untreated **(A)** or CQ-treated **(B)** showing parasite load in liver 40 h after injection of 1.5 × 10^4^ *Py*-Luc salivary gland SPZ. Rainbow scale represents radiance (p/s/cm^2^/sr). **(C)** Quantification of total flux from whole body imaging of mice (shown in **A** and Supplementary Figure S1). *n* = 3 for SPZ ( infected and untreated) and NI (neither infected nor treated) group, four for SPZ+CQ group and five each for SPZ+PQ and drug control (treated uninfected) groups. **(D)** Quantification of LS parasite burden 40 hpi in livers of SPZ (*n* = 3), SPZ+CQ (*n* = 4), and SPZ+PQ (*n* = 5) mice by qPCR. Graph represents Py-18S RNA normalized to murine β-actin RNA. Data in **(C,D)** represents Mean ± SD, Mann–Whitney test was performed and *p* < 0.05 considered as significant. CQ, Chloroquine; PQ, Primaquine; NI, non-infected and non-treated.

*Plasmodium* undergoes extensive multiplication during LS development, producing tens of thousands of merozoites from an individual sporozoite. Upon completion of LS development, merozoites are released into the blood stream as small merozoite-filled vesicles called merosomes. To quantify the multiplication and subsequent release of parasites in the liver, we infected mice with 1.5 × 10^4^ luciferase expressing *Py*-Luc SPZ and performed BLI at 40, 44, 48, 54, and 62 hpi. Parasite biomass increased with time until 44 h and then gradually declined with the lowest LS parasite burden at 54 hpi (**Figures 1** and **2**; Supplementary Figure S2). We could detect measurable luciferase activity in the liver at 54 hpi with an average total flux of 5.0 × 10^6^ photon/s (**Figure 1**). By 62 hpi, parasites are detected throughout the body, indicating a blood stage infection (Supplementary Figure S1E), and any residual parasites in the liver could not be distinguished from circulating blood stage parasites.

### CQ Neither Kills LS Parasites Nor Delays Their Development

**FIGURE 3:**
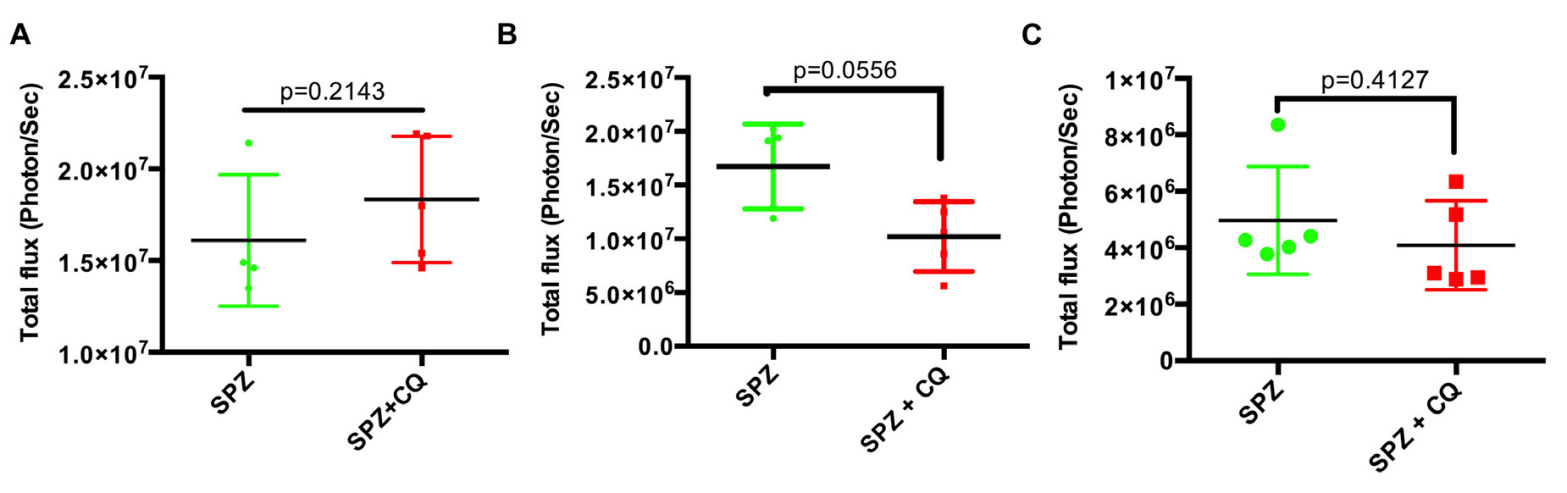
**Parasite load in liver at different time points after CQ treatment.** Quantification of total flux from whole body imaging of mice (shown in Supplementary Figure S4). *n* = 4 for SPZ group at 44 hpi **(A)**, five each for both SPZ and SPZ+CQ at 48 h **(B)**, and 54 h **(C)** time points. Graph represents mean ± SD. Mann–Whitney test was performed and *p* < 0.05 considered as significant.

To evaluate the effect of CQ on developing LS parasites, we infected mice with 1.5 × 10^4^ *Py*-Luc SPZ, and then treated with either CQ (0.8 mg/mouse), PQ (1.5 mg/mouse), or an equal volume of PBS. Three groups of uninfected mice received same treatments (CQ, PQ, or PBS) but no SPZ. Imaging of whole bodies or of isolated livers was performed at 40, 44, 48, and 54 hpi. CQ-treated (SPZ+CQ) and untreated (SPZ) mice did not differ significantly at any time point (**Figures 2** and **3**; Supplementary Figure S2), whereas the SPZ+PQ group was completely devoid of any bioluminescence, as expected (**Figure 2**; Supplementary Figure S1). Parasite burden estimation with conventional qPCR gave similar results (**Figure 2D**). At 40 hpi, bioluminescence appeared lower in SPZ+CQ group than SPZ group but this trend was not statistically significant (*p* = 0.06; **Figure 2C**), and no such trend was observed in the corresponding qRT-PCR measurements at this time point (*P* = 0.86; **Figure 2D**).

**FIGURE 4:**
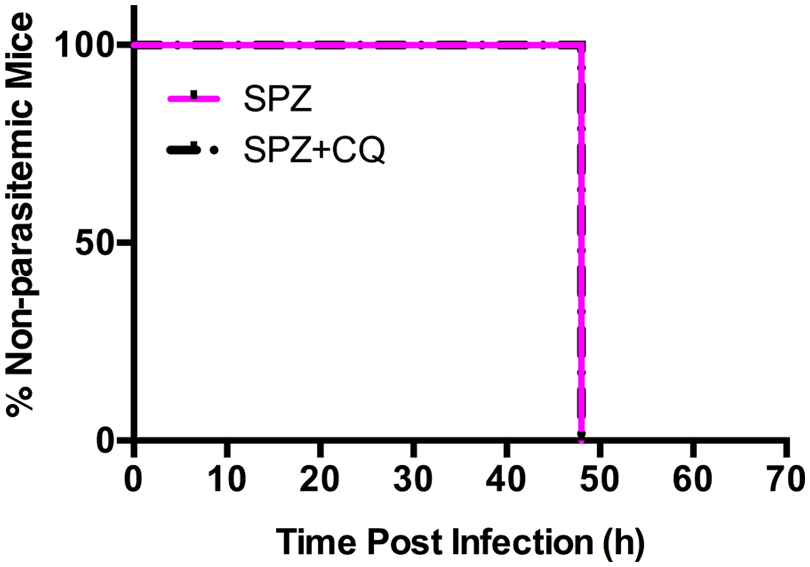
**Effect of CQ on prepatent period of *Py*-Luc.** Mice (*n* = 18) were infected with 1.5 × 10^4^ *Py*-Luc salivary gland SPZ and either CQ-treated (SPZ+CQ; *n* = 9) or not (SPZ; *n* = 9). Blood smears (both thick and thin smears) were collected starting from 48 hpi. Smears were stained and examined by expert smear reader blinded. Smears are considered positive if minimum two parasites were found in 100 adjacent fields.

To remove inter-subject variability in a second experiment, we followed individual mice over time, and acquired BLI at sequential time points. We infected each of 20 mice with SPZ of *Py*-Luc; half were treated with CQ while half received an equal volume of PBS. At 42, 44, 46, and 48 hpi, LS parasite burden was measured by whole body BLI. We did not observe significant differences in the parasite burden in the SPZ+CQ *versus* SPZ animals at any time point (Supplementary Figures S3 and S4). In a third experiment, using parental Py-17XNL infection, we confirmed by qPCR that the LS parasite burden at 48 hpi does not significantly differ between SPZ+CQ and SPZ animals (*p* = 0.38, Supplementary Figure S5). Both SPZ+CQ and SPZ mice became blood stage positive at 48 hpi by blood smear microscopy, further indicating that CQ does not delay LS development (**Figure 4**).

## Discussion

The effect of CQ on *Plasmodium* blood stages is well characterized, with the inhibition of heme-polymerase enzyme leading to accumulation of toxic heme products, and parasite death (Slater and Cerami, 1992). CQ-CVac generates strong LS-specific immune responses and more durable protection at a log-fold lower SPZ dose as compared to RAS immunization (Clyde et al., 1973; Bijker et al., 2013). However, it is not clear how CQ treatment helps generate this strong and durable immune response. One school of thought holds that CQ-CVac allows the complete development of LS parasites that express a wide array of LS specific antigens, prior to killing the parasites in their first cycle of blood stage development; however, untreated *Plasmodium* infections also display the full antigen repertoire without inducing sterile preerythrocytic immunity. We hypothesized that CQ may have partial or subtle effects on late LS development that contribute to enhanced immune responses, but we find no evidence that CQ decreases LS burden or delays LS development.

The effect of CQ on the LS of *Plasmodium* has previously been studied *in vivo* in a mouse model (Belnoue et al., 2004) and *in vitro* by infection of non-human primate hepatocytes (Fisk et al., 1989). Belnoue et al. (2004) reported that mice treated with 0.8 mg CQ and untreated mice had similar LS parasite burden 42 hpi, but did not examine effects beyond this time point; the CQ dose (∼32 mg/kg) used by Belnoue et al. (2004, and also by us for this study) was based on earlier work (Orjih et al., 1982), and is in the dosing range (30–35 mg/kg) used for children of 10–20 kg body weight (Moore et al., 2011). Earlier, Fisk et al. (1989) reported that CQ at 1 μg/ml had a partial parasiticidal effect *in vitro* on late LS *P. cynomolgi* and *P. knowlesi* schizonts. However, these drug concentrations were ∼2.5-fold higher than therapeutic concentrations (0.03–0.4 μg/ml) in humans (Tett et al., 1989; Carmichael et al., 2003), and had toxic effects on hepatocytes that may have contributed to parasiticidal activity.

We examined the effect of CQ on LS development *in vivo* by imaging luciferase expressing *P. yoelii* parasites in Balb/c mice, and followed parasite development in the liver, both by BLI and qPCR, at multiple time points from 40 h through 62 h. We observed no difference in the LS parasite load between CQ-treated (SPZ+CQ) and untreated (SPZ) groups at any time point during late LS development. Our observations at 42 hpi are consistent with those of Belnoue et al. (2004) and additionally showed no differences at 44, 46, 48, and 54 hpi.

We hypothesized that CQ might delay LS development, at least for a subset of parasites, thereby creating a condition of prolonged antigen exposure that might enhance LS specific immunity. All mice, both SPZ+CQ and SPZ, had parasites detectable by blood smear microscopy by 48 hpi, suggesting that there had been no delay in LS development with CQ treatment. This was further substantiated by similar LS parasite burdens in both groups of mice at 48 hpi (**Figure 3**; Supplementary Figures S3–S5). Our study cannot exclude the possibility that a small subset of parasite persist in liver of SPZ+CQ mice, because emerging blood stage parasites might mask the presence of LS forms in bioluminescence or qRT-PCR studies.

The potential effect of CQ to delay the prepatent period has also been examined in humans as part of CVac trials, using highly sensitive qPCR techniques to detect, and quantify blood stage parasites at densities below the sensitivity of microscopy (i.e, subpatent parasitemia; Roestenberg et al., 2009; Bijker et al., 2013). During CVac, blood stage parasites are detected by qPCR at the time expected of untreated individuals (Roestenberg et al., 2009; Bijker et al., 2013), although a direct comparison of blood stage parasite burden between CQ-treated and untreated individuals has not been reported. Similarly, we find that blood stage parasites are detected by microscopy at 48 hpi in both SPZ+CQ and SPZ mice. A previous study in mice reported that CQ suppressed blood stage growth during CVac (Peng et al., 2014), as is observed in human studies (Roestenberg et al., 2009; Bijker et al., 2013). Our results in mice confirm that CQ does not delay LS development nor kill LS parasites, and support the notion that the diminished first wave of parasitemia is likely to be solely due to its effects on blood stage growth, rather than the effect on the number of parasites released from liver.

Chloroquine was once widely used as an anti-malarial drug and was highly effective in clearing blood stage forms of sensitive *Plasmodium* parasites, but use of CQ has waned decisively with the global spread of CQ-resistant parasites. Recently, the impressive immunity observed after CVac has sparked renewed interest in CQ as a component of whole organism vaccines, whereby individuals are inoculated with infectious SPZ under CQ drug cover. However, the mechanisms by which drug treatment converts infectious SPZ into an effective vaccine remain unclear. Using a luciferase-expressing parasite and an *in vivo* mouse infection model, we have systematically evaluated the effect of CQ on *Plasmodium* late liver stages. We report that CQ given at a dose used for CVac studies neither kills nor delays LS parasite development. However, our study does not exclude other effects of CQ on *Plasmodium* LS biology that might impact immune responses, such as an altered repertoire of expressed antigens, and hence further investigation is warranted.

## Author Contributions

Conceived and designed the experiments: TS and PD. Performed the experiments: TS, LL, JH, SC, SO-G, and DC. Analyzed the data: TS and PD. Wrote the manuscript: TS and PD.

## Conflict of Interest Statement

The authors declare that the research was conducted in the absence of any commercial or financial relationships that could be construed as a potential conflict of interest.
