# Supplementary material for: Chloroquine neither eliminates liver stage parasites nor delays their development in a murine Chemoprophylaxis Vaccination model
Source: Front Microbiol. 2015 Apr 9;6:283. doi: 10.3389/fmicb.2015.00283 (PMC4391028; doi:10.3389/fmicb.2015.00283)
Supplement: Supplementary file 1 [file image_1.pdf]

Supplementary Material

Chloroquine neither eliminates liver stage parasites nor delays their development in a murine Chemoprophylaxis Vaccination model

Chloroquine and *Plasmodium* liver stage

Tejram Sahu,[1*] Lynn Lambert,[1] Jessica Herrod,[1] Solomon Conteh,[1] Sachy Orr-Gonzalez,[1] Dariyen Carter[1], Patrick E. Duffy[1*]

[1] Laboratory of Malaria Immunology and Vaccinology, National Institute of Allergy and Infectious Diseases, National Institutes of Health, Rockville, Maryland, USA

Correspondence:
*Dr. Patrick E. Duffy
Laboratory of Malaria Immunology and Vaccinology
National Institute of Allergy and Infectious Diseases
National Institutes of Health
5640 Fishers Lane, RM-1111
Rockville, MD, 20852, USA
Phone: +1 301-443-4605
Fax: +1 301-480-1958
E-mail: duffype@niaid.nih.gov

*Dr. Tejram Sahu
Laboratory of Malaria Immunology and Vaccinology
National Institute of Allergy and Infectious Diseases
National Institutes of Health
12735 Twinbrook Parkway, RM-3W15
Rockville, MD, 20852, USA
Phone: +1 301-402-0158
E-mail: sahut@mail.nih.gov

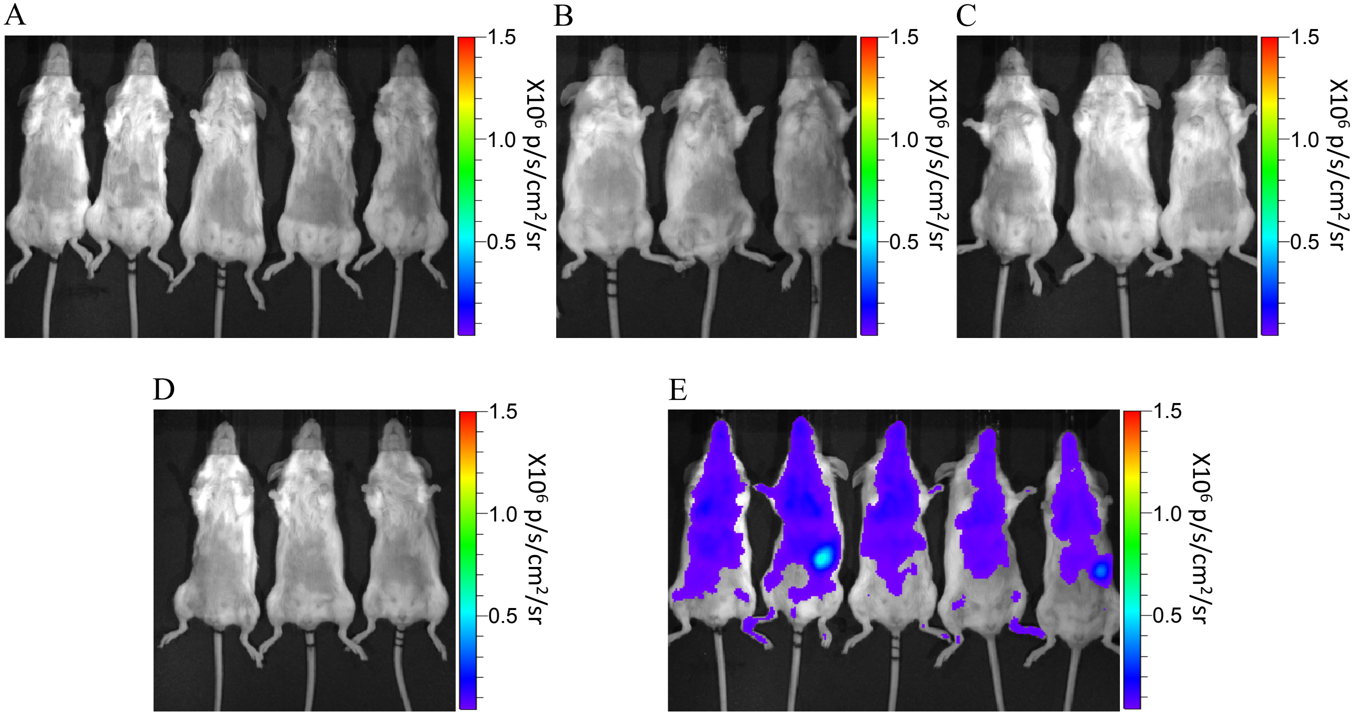

A
B
C
D
E
1.5
1.0
0.5
X10^6 p/s/cm^2/sr

Figure S1. Parasite load of control mice measured by BLI. Rainbow images of SPZ (A) SPZ+PQ, (B) CQ, (C) PQ, and (D) NI mice after either injection of $1.5x10^4$ Py-Luc salivary gland SPZs or only drug treatments. Bioluminescence data is presented in Figure 2 panel C. (E) Rainbow image of control mice at 62 hpi showing blood stage infection. Rainbow scale represents radiance (p/s/cm^2/sr).

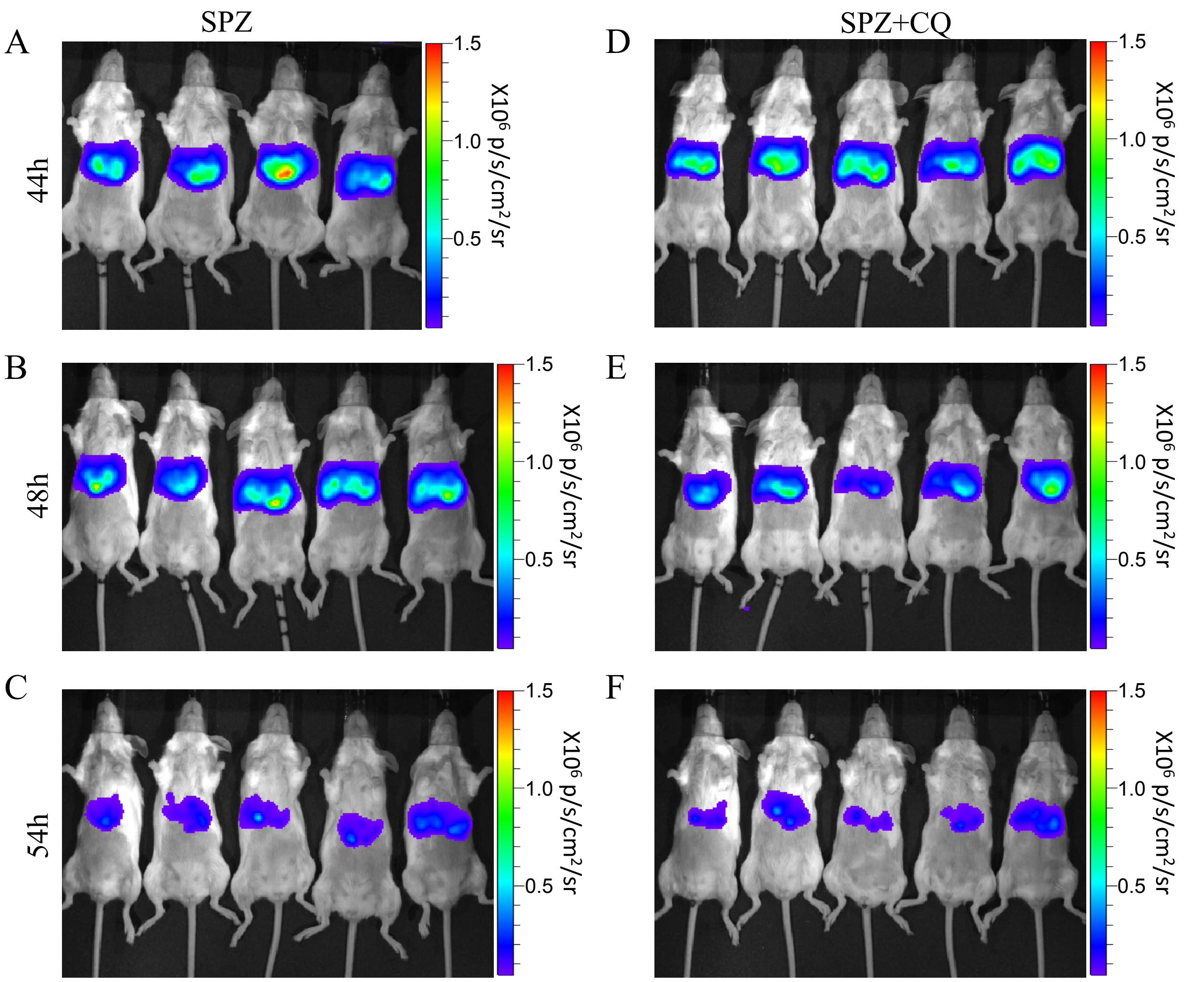

SPZ
SPZ+CQ
A
B
C
D
E
F
44h
48h
54h
1.5
1.0
0.5
X10^6 p/s/cm^2/sr

Figure S2. BLI of CQ-treated and control mice at 44 through 54hpi. Rainbow images of mice infected with $1.5x10^4$ Py-Luc salivary gland SPZs and either treated with CQ (0.8 mg/mouse) (D, E and F) or PBS (A, B and C). Images were acquired at (A and D) 44h, (B and E) 48h and (C and F) 54h. Rainbow scale represents radiance ($p/s/cm^2/sr$). BLI data for these images were presented in Figure 3.

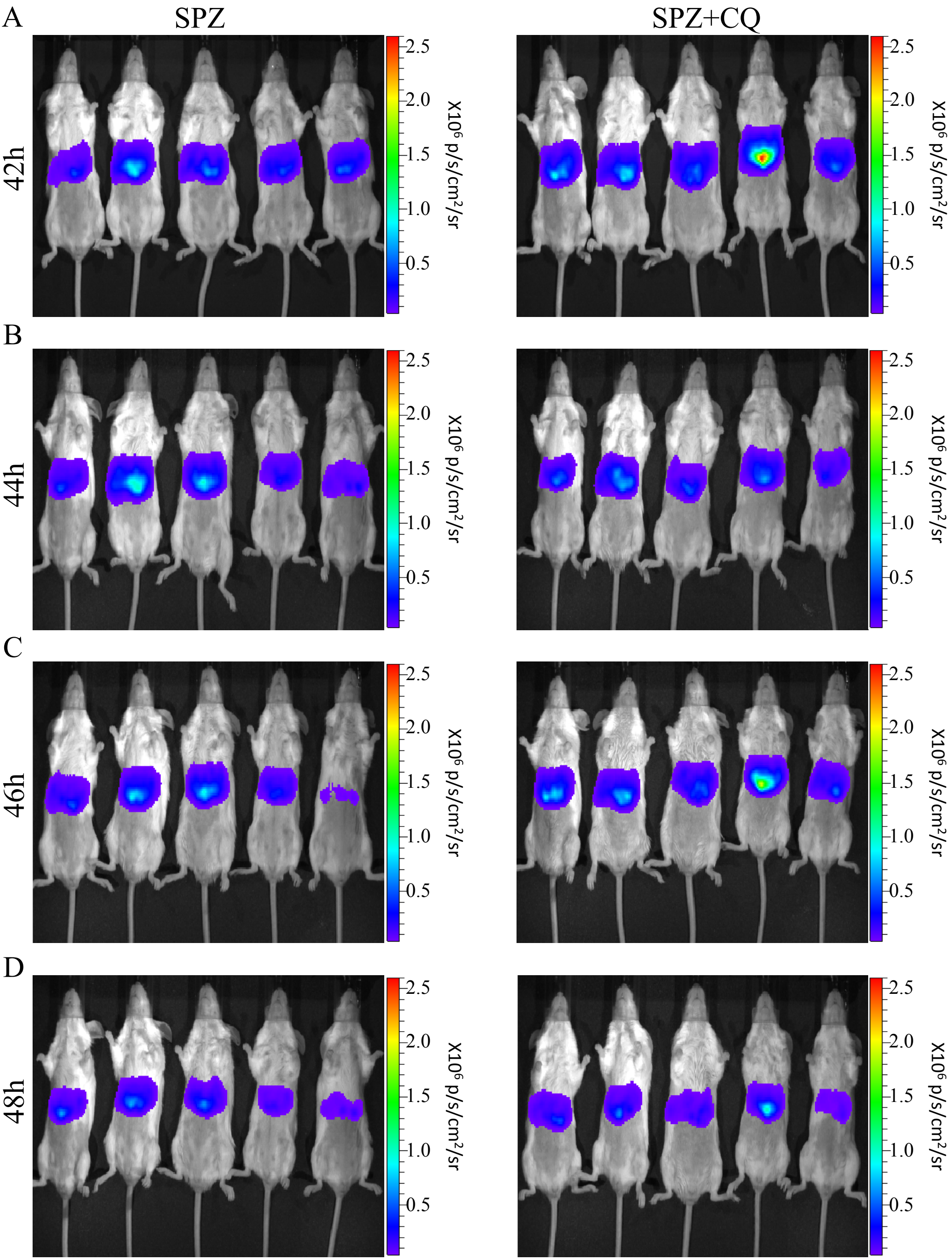
A
SPZ
SPZ+CQ
42h
B
44h
C
46h
D
48h
2.5
2.0
1.5
1.0
0.5
X10^6 p/s/cm^2/sr

Figure S3. LS development upon CQ treatment followed from 42h through 48h by BLI. Representative rainbow images of mice infected with 1.5×10^4 Py-Luc salivary gland SPZs and either treated with CQ (0.8 mg/mouse) (right panel) or PBS (left panel). Images were acquired at (A) 42h, (B) 44h, (C) 46h, and (D) 48h. Rainbow scale represents radiance ($p/s/cm^2/sr$). BLI data for these images were presented in Figure S4.

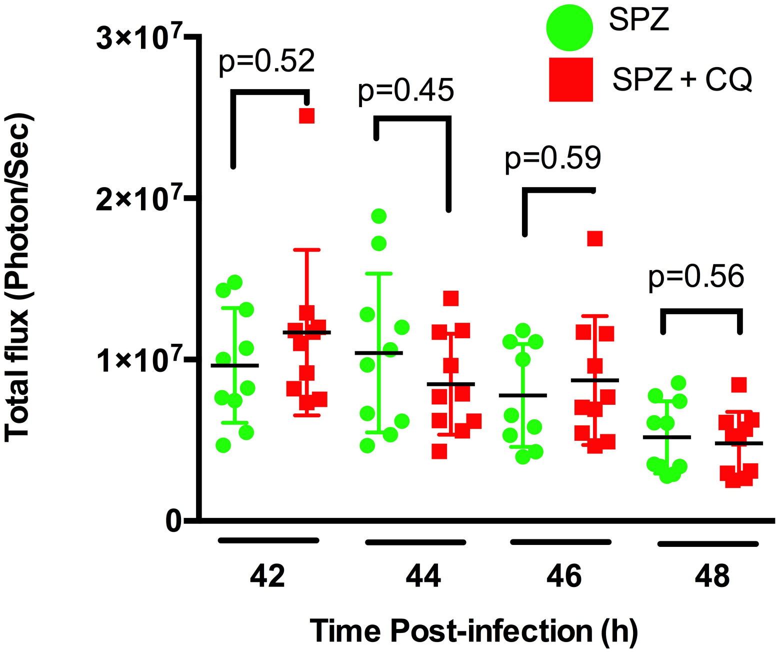

SPZ
SPZ + CQ
p=0.52
p=0.45
p=0.59
p=0.56
3×10^7
2×10^7
1×10^7
0
Total flux (Photon/Sec)
42
44
46
48
Time Post-infection (h)

Figure S4 Continuous monitoring of the effect of CQ on *Plasmodium* LS development. Quantification of total flux from whole body imaging of mice (shown in Figure S5). Mice (n=20) were infected with 1.5×10^4 Py-Luc salivary gland SPZ and either CQ-treated (n=10) or not (n=10). Images were acquired every 2h from 42 hpi until 48 hpi. Graph represents mean ± SD. Mann-Whitney test was performed and $p<0.05$ considered as significant.

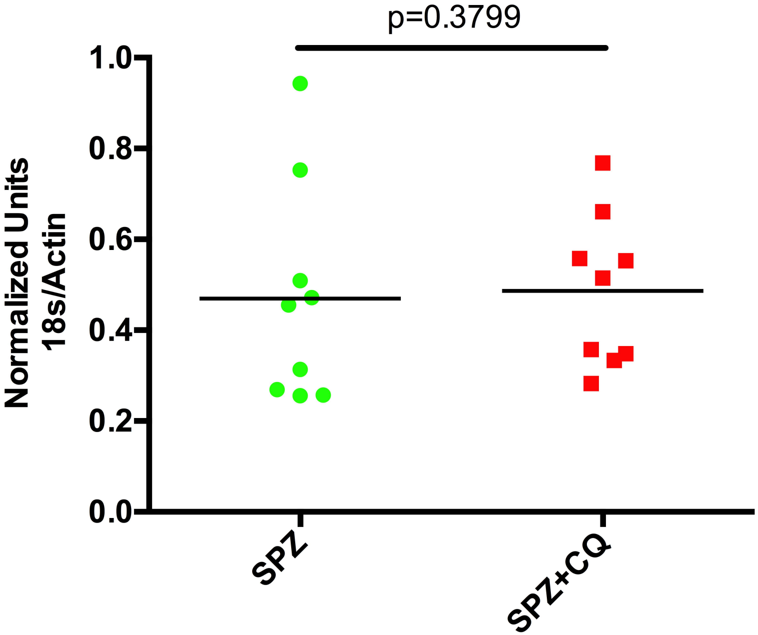
p=0.3799
Normalized Units
18s/Actin
1.0
0.8
0.6
0.4
0.2
0.0
SPZ
SPZ+CQ

Figure S5. LS Parasite burden at 48hpi. Quantification of LS parasite burden by qPCR. Mice (n=18) infected with $1.5x10^4$ Py-17XNL SPZs, one group (n=9) received CQ (0.8 mg/mouse) at 0 and 24hpi, another group (n=9) received equal volume of PBS. Graph represents Py-18S RNA normalized to murine β-actin RNA. Data represents normalized Mean ± SD, Mann-Whitney test was performed and $p<0.05$ is considered as significant.
